# Ensemble Learning for Spatial Interpolation of Soil Potassium Content Based on Environmental Information

**DOI:** 10.1371/journal.pone.0124383

**Published:** 2015-04-30

**Authors:** Wei Liu, Peijun Du, Dongchen Wang

**Affiliations:** 1 Jiangsu Provincial Key Laboratory of Geographic Information Science and Technology, Nanjing University, Nanjing, Jiangsu, People’s Republic of China; 2 School of Geodesy and Geometrics, Jiangsu Normal University, Xuzhou, Jiangsu, People’s Republic of China; Xiamen University, CHINA

## Abstract

One important method to obtain the continuous surfaces of soil properties from point samples is spatial interpolation. In this paper, we propose a method that combines ensemble learning with ancillary environmental information for improved interpolation of soil properties (hereafter, EL-SP). First, we calculated the trend value for soil potassium contents at the Qinghai Lake region in China based on measured values. Then, based on soil types, geology types, land use types, and slope data, the remaining residual was simulated with the ensemble learning model. Next, the EL-SP method was applied to interpolate soil potassium contents at the study site. To evaluate the utility of the EL-SP method, we compared its performance with other interpolation methods including universal kriging, inverse distance weighting, ordinary kriging, and ordinary kriging combined geographic information. Results show that EL-SP had a lower mean absolute error and root mean square error than the data produced by the other models tested in this paper. Notably, the EL-SP maps can describe more locally detailed information and more accurate spatial patterns for soil potassium content than the other methods because of the combined use of different types of environmental information; these maps are capable of showing abrupt boundary information for soil potassium content. Furthermore, the EL-SP method not only reduces prediction errors, but it also compliments other environmental information, which makes the spatial interpolation of soil potassium content more reasonable and useful.

## Introduction

High quality soil property maps based on spatial patterns of soil variability are needed for agricultural planning, risk assessments, and decision making in regards to environmental management and conservation. However, such maps are usually not readily available and they are often difficult and expensive to acquire, especially for mountainous and high altitude regions. Furthermore, sampling points for soil potassium content are typically sparse and the available data may be insufficient to characterize the highly variable soil potassium content and its spatial patterns. Therefore, it would be worthwhile to develop methods that can estimate soil potassium content in areas where soil potassium content has not been measured. Spatial interpolation, which is method that can be used to construct continuous data from variables measured at point locations, is a promising technique for soil potassium content studies[1].

Three main limitations are typically encountered when using spatial interpolation techniques to estimate soil properties. These limitations include small numbers of available soil samples, nonlinearity of the relationships between environmental variables and soil properties, and the optimal spatial interpolation model for a given study region is often not known. When sampling points are sparse or poorly correlated in space, it can be difficult to interpolate the distributions of soil properties accurately without secondary environmental variables [2–5]. Some studies have indicated that important relationships exist between soil properties and different types of geographical information, including terrain attributes derived from digital elevation models (DEM) [6–9], and the distributions of land use types, rock types, soil types, etc., [10], and other soil attributes related to target variables[11].

Many interpolation methods can be used to process soil potassium content data. Non-geostatistical methods such as inverse distance weighting (IDW) with an interpolator assume that each input point has a local influence that diminishes with distance and no additional assumptions are required for the data[12]. The IDW technique is commonly applied because of its relative simplicity and availability. However, predictions from IDW are usually associated with large errors. Geostatistical methods like kriging assume that the distances and directions between sample points contain spatially correlated data that can be used to explain variation in the surface [13]. The kriging interpolation model provides the best linear unbiased estimates, accurate descriptions of the spatial structure of data, and valuable information about the estimation error distributions [14]; while it is usually better than IDW, kriging is based on a model whose assumptions (e.g., stationary hypothesis) may not be met in practice.

The interpolation methods discussed so far often need data that meet certain conditions or are parameter-specific, and the performances of the methods can be influenced by many factors [4]. These influencing factors are problematic in that no consistent findings have been obtained on them to date, which makes it challenging to select an optimal method for spatial interpolation. Therefore, it is often difficult to select an appropriate spatial interpolation method for a given study area.

In recent years, some machine learning methods have been applied to the fields of data mining and spatial interpolation, and these studies have demonstrated the utility of such methods in terms of data accuracy and processing efficiency. e.g., random forest(RF), support vector machine (SVM), and some other learning methods [8, 15–25]. Furthermore, SVM has been applied to rainfall data in a study by Gilardi [26]. Notably, ensemble learning has not been applied yet to the spatial interpolation of soil properties.

The specific objectives of this study are (1) to describe spatial distributions of soil properties accurately based on soil types, geology types, land use types, and slope by applying ensemble learning with ancillary environmental information for improved interpolation of soil properties (hereafter, EL-SP) and (2) to compare the performance of the EL-SP method with IDW, universal kriging (UK), ordinary kriging (OK), and a method that combines OK with different types of geographic information. To accomplish these objectives, we applied the EL-SP method along with the other interpolation methods to soil potassium content data we collected from around Qinghai Lake in China during September 2013. The prediction patterns of the interpolation methods were analyzed based on their prediction maps.

## Methods

### EL-SP

As a modified versions of regression kriging [9, 27], each observation *z*(*xi*,*yj*) of one specific soil potassium content at grid (*i*,*j*) can be expressed as:

$$z(x_{i},y_{j})=t(x_{i},y_{j})+r(x_{i,l,k},y_{j,l,k})$$ (1)

where *t*(*xi*,*yj*) is the trend value of *z*(*xi*,*yj*) in the grid (*i*,*j*) and *r*(*xi*,*l*,*k*,*yj*,*l*,*k*) is the residual in the *l*th type of *k*th environmental information computed by subtracting the trend value of *t*(*xi*,*yj*) from the measured value of soil potassium content. We assumed that *t*(*xi*,*yj*) and *r*(*xi*,*l*,*k*,*yj*,*l*,*k*) are independent of each other and the variation of *r*(*xi*,*l*,*k*,*yj*,*l*,*k*) is homogeneous over the overall study area.

The residuals of the relevant types of environmental information were then used to interpolate the surface of residuals in the whole study area by ensemble learning. The interpolated values of residuals were finally summed to the soil potassium content trend as the final interpolated values of EL-SP interpolation.

**Fig 1:**
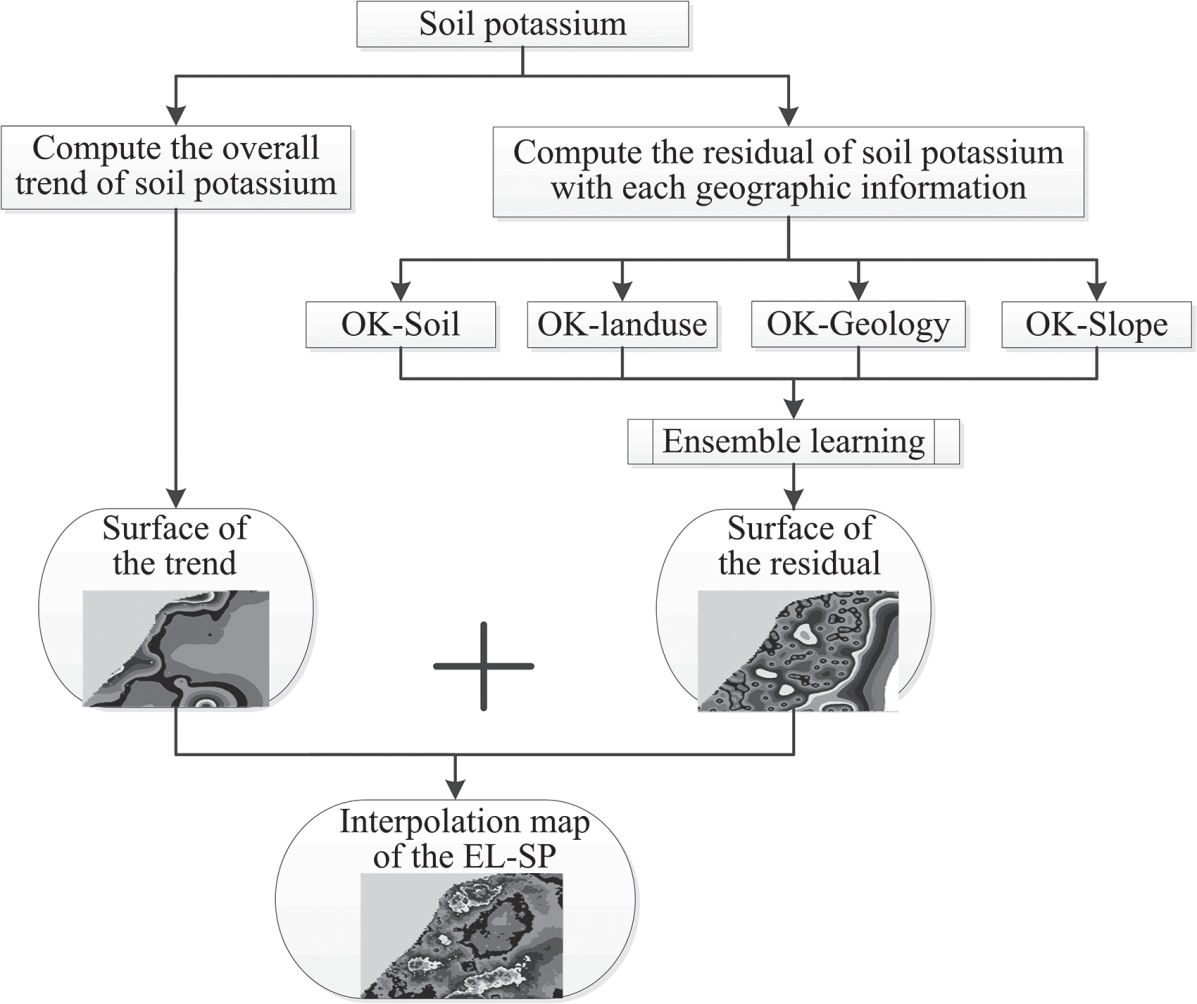
Framework of EL-SP method.

The framework of EL-SP (Fig 1), and the processes used were as follows.

1. With the measured soil potassium content values, we calculated the trend and residuals of soil potassium content.
2. According to the spatial distribution of the soil potassium content trend value, we mapped the overall distribution of soil potassium content *t* (*xi*,*yj*) for each grid (*i*,*j*).
3. According to the related residuals, we used OK combined with soil type (OK-Soil), OK combined with geology type (OK-Geology), OK combined with land use type (OK-Landuse), and OK combined with slope type (OK-Slope) for the remaining residual interpolation.
4. Ensemble learning was then used to integrate all the residual surfaces (OK-Soil, OK-Geology, OK-Landuse, and OK-Slope) to obtain the soil potassium content *r*(*xi*,*l*,*k*,*yj*,*l*,*k*).
5. Finally, we added up the trend surface and residual surface for the EL-SP simulation results.

Typically, an ensemble is constructed in two steps. First, a number of base learners are produced (e.g., OK-Landuse, OK-Soil, OK-Geology, and OK-Slope). Then, the base learners are combined for use, where the most popular combination schemes are used in majority voting for classification and weighted averaging for regressions. The pseudo-code of ensemble learning is as below:

**Input:**

// *x*_*i*_ is measured value, *X* is measured values set; y_*i*_ is predicted value, *Y* is predicted values set.

Data set D = {(*x*_*1*_, *y*_*1*_), (*x*_*2*_, *y*_*2*_)⋯ (*x*_*m*_, *y*_*m*_)} where, *x*_*i*_∈*X*, *y*_*i*_∈*Y*;

// *h* is the interpolation model set (e.g., OK-Landuse, OK-Soil and OK-Geology) 

Base learner *h;*

Number of learning rounds *n*.

**Process:**

*for t = 1，*…,*n;*

//Measure the interpolation error of *h*_*t*_ $\varepsilon_{t}=\frac{\sum_{i=1}^{n}\frac{\left|y_{i}-x_{i}\right|}{x_{i}}}{n}$

*if ε*_*t*_≥*1/2*, then stop;

// Determine the weight of *h*_*t*_

*Set* $a_{t}=\frac{1}{2}\text{ln}(\frac{1-\varepsilon_{t}}{\varepsilon_{t}})$

end

**Output:**

The final interpolation function: $H(\text{x})=\sum_{t=1}^{\text{n}}z_{t}h_{t}(x)$

Where *Z*_*t*_ is a normalization factor: $z_{i}=\frac{a_{i}}{\sum_{i=0}^{n}a_{i}}$

### Parameter specification and secondary variable selection

The specification of parameters was based on the requirements of the interpolation methods and data characteristics. The interpolation methods were performed using the geostatistics analysis and 3D analyst modules of ArcGIS 10.1. Different parameters for IDW, UK, OK, and OK-Geo (OK with combined geographic information) were compared, and the optimal parameters with the smallest root mean square error (RMSE) values were determined. For the OK and its combined methods, the spherical, exponential, Gaussian, and linear models were fitted to the experimental variogram, and the number of the closest samples chosen varied from 5 to 30 with five-step intervals. The IDW was estimated with powers of 1, 2, 3, and 4.

In order to analyze which secondary variables were significant for soil potassium content, the analysis of variance (ANOVA) procedure was used to test for the significance of geographic type effects on the variances of soil potassium content (Table 1). Table 1 shows that land use types, soil types, geology types, and slopes were the four most strongly correlated variables with soil potassium content based on the results of the ANOVA analysis; these variables were all significant at the 0.01 level, and they were used as secondary variables in EL-SP and OK-Geo. The OK and IDW methods do not need secondary variables.

**Table 1:** The ANOVA results for testing the secondary variables effects on the variances of soil potassium content.

Method	Secondary variables	Source of variance	Sum of squares	df	Mean square	F	Sig.
EL-SP	Land use types	Between	1.323	5	0.243	7.342	0.000
OK-Geo	Land use types	Within	5.245	145	0.033		
		Total	6.568	150			
EL-SP	Soil types	Between	1.542	6	0.261	6.781	0.000
OK-Geo	Soil types	Within	5.026	144	0.038		
		Total	6.568	150			
EL-SP	Geology types	Between	1.524	11	0.278	7.512	0.000
OK-Geo	Geology types	Within	5.044	139	0.037		
		Total	6.568	150			
EL-SP	Slope	Between	1.451	4	0.236	5.250	0.000
OK-Geo	Slope	Within	5.117	146	0.045		
		Total	6.568	150			

## Study Area and Dataset

### Study area

**Fig 2:**
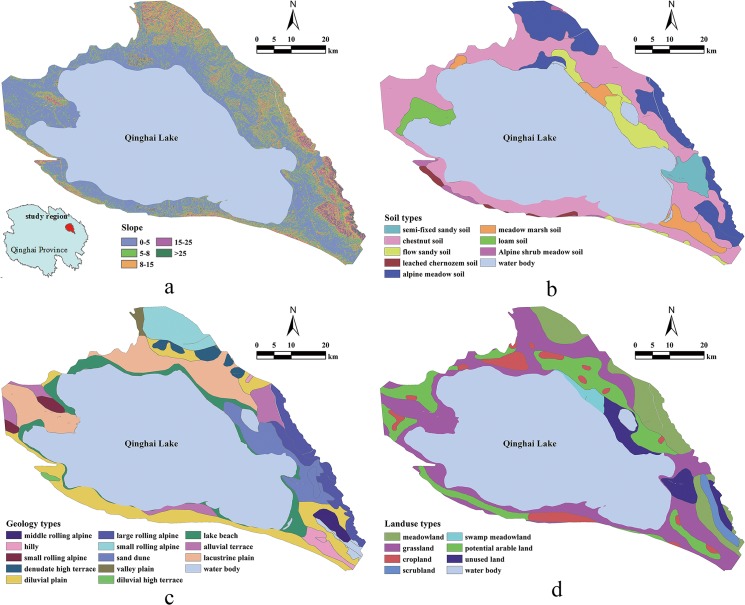
Environmental information of the study area. Types of (a) slope, (b) soil, (c) geology, and (d) land use.

The study area (36°32′56″–37°48′40″N, 99°51′29″–101°12′82″E) is located in the northwest region of the Qinghai Province in China (Fig 2A). This region covers an area of 8753.73 km^2^, and a large portion of this area is covered with water (4473.96 km^2^). The region comprises three geomorphic counties including Gangcha, Haiyan, and Gonghe counties. The study area is part of the Qinghai Lake basin, and the elevation is varies from 2008 to 4616 m. According to 1:1000, 000 scale soil maps reported by the National Soil Census Office, there are 9 soil types in this region, and these soil types include alpine meadow soil, semi-fixed sandy soil, meadow marsh soil, alpine shrub meadow soil, chestnut soil, and leached chernozem soil, etc. (Fig 2B). According to the 1:500,000 geologic map of the Qinghai Province from the Qinghai Provincial Bureau of Geology and Mineral Resources, the regional geology types include valley plain, alluvial terrace, lake beach, and denudation high terrace, etc. (Fig 2C). Regional land use types were classified into water body, croplands, grasslands, swamp meadow land, shrub land, and unused lands, etc. (Fig 2D). According to the correlation between slope and soil potassium content, the slopes were classified into five groups including 0°–5°, 5°–8°, 8°–15°, 15–25°, and >25° (Fig 2A).

### Dataset

We collected a total of 193 topsoil samples (0–30 cm) from the Qinghai Lake region in September 2013(S1 Dataset). The sampling activities were approved orally by members of the Environmental Monitoring Center of the Qinghai Province, who were project participants. At the sampling sites, we recorded the locations of the soil samples, the elevation, soil types, geology types, and land use types. Each sample was air-dried and passed through a 2 mm sieve prior to determining the soil potassium contents used in this study.

A large amount of environmental information can be used as secondary variables to improve the performance of spatial interpolation methods as discussed by Li and Heap [4] and Shi et al[5]. After preliminary analyses, soil types, geology types, land use types, and slope data were considered as important secondary information in this study. Soil types, geology types, and land use types have been used in earlier work to improve the performance of spatial interpolators of soil properties [5], so the inclusion of this type of environmental information was expected to improve the prediction accuracy. Slopes are likely to have some influence on the transfer of soil potassium content from high slope regions to low slope regions, so slope was also considered as an important secondary variable in this study with the potential to improve the overall prediction accuracy. Since the relationships between the soil potassium content and the secondary information variables were nonlinear, ANOVA analysis was used measure the correlations: the soil potassium content displayed a significant correlation with land use types (*f* = 7.342, *p*-value = 0.000), soil types (*f* = 6.781, *p*-value = 0.000), geology types (*f* = 7.512, *p*-value = 0.000), and slope types (*f* = 5.250, *p*-value = 0.000).

The dataset of environmental information was generated in ArcGIS 10.1, and where necessary, the data were resampled to a 30 m resolution. However, the limited soil potassium content sample sizes and uneven distribution of sample points meant that there were some geographic areas without enough sample data for modeling; hence, we tried to use the environmental variables to improve the spatial interpolation accuracy in such areas. The soil potassium content samples were divided into two groups. One was the training subset with 150 samples, and the other was the test subset with 43 samples. We used the training subset to estimate values of the test subset, and then, we compared the predicted and measured values for every data point of the test subset. The mean error (ME), mean absolute error (MAE), and RMSE were calculated using the predicted and measured values at each validation sample site for the test subset.

## Experiments and Discussion

### Accuracy of EL-SP

In order to assess the accuracy of EL-SP for interpolating soil potassium contents, we compared the performance of the proposed EL-SP method to the UK, OK, IDW, and OK-Geo techniques. The ME, MAE, and RMSE values, which were calculated using the predicted and measured values, are shown in Table 2; these values reflect the interpolation quality of the different methods. We found that the interpolators that were combined with environmental information, i.e., EL-SP and OK-Geo, were the most accurate methods. Additionally, the OK and IDW methods outperformed the UK method. The combination of environmental information with OK considerably improved the prediction accuracy, although the resulting values were less accurate than those from EL-SP. The higher accuracy of EL-SP is thought to be related to its good ability to discern boundaries among the different geographic features, which in turn led to more accurate descriptions of the spatial variation characteristics of soil potassium content in different geographic types.

**Table 2:** Comparisons of the accuracy among the UK, OK, IDW, OK-Geo, and EL-SP methods.

Methods	ME ^a^	MAE ^a^	RMSE ^a^
IDW	-0.004076	0.158753	0.197244
UK	-0.001331	0.161025	0.216935
OK	0.003384	0.158505	0.198844
OK-GEO	0.000129	0.132403	0.193699
EL-SP	0.000125	0.131042	0.190325

^a^ ME, mean error; MAE, mean absolute error; RMSE, root mean square error.

### Maps of EL-SP

**Fig 3:**
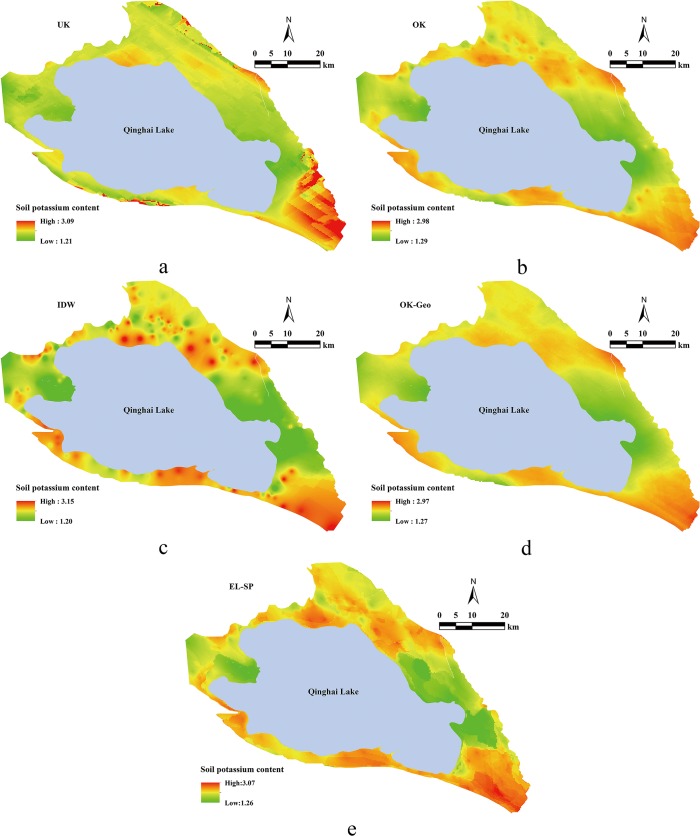
Comparisons of the soil potassium content maps interpolated by (a) UK, (b) OK, (c) IDW, (d) OK-Geo, and (e) EL-SP.

The spatial predictions of soil potassium content for the five methods (i.e., UK, OK, IDW, OK-Geo, and EL-SP) are illustrated (Fig 3). The spatial distribution patterns of the two most accurate methods (i.e., EL-SP and OK-Geo) were similar and they captured the major spatial distribution patterns and trends of soil potassium content; but weak “bull’s eyes” patterns were evident with OK-Geo. The ranges of results were somewhat narrower in the predictions. The predictions of UK (Fig 3A) produced a map with linear tracks, sharp transitions, and banding patterns. The predictions of OK (Fig 3B) produced a map similar to that of OK-Geo and EL-SP, but with the sharp transitions and evident “bull’s eyes” patterns. The predictions of IDW (Fig 3C) reproduced the major patterns, but it failed to predict changes in local variation and displayed strong “bull’s eyes” patterns at sample points with either high or low values. In OK-Geo (Fig 3D) and EL-SP (Fig 3E), the combined environmental information helped to eliminate the linear tracks, sharp transitions, and banding pattern effects that were apparent in the OK and UK maps. Overall, these results suggest that interpolators that combine data with additional environmental information can describe the local variation more accurately. Additionally, such techniques show great promise for improving the interpolation performance in difficult to study regions.

Although the proposed EL-SP method was found to produce more accurate results than the other traditional spatial interpolation methods that were tested, the search for optimized statistical spatial interpolation methods is still in its early stages. Other combined approaches that use machine learning methods with existing spatial interpolation techniques may also yield valuable results. In particular, learning methods such as distribution learning, Hausdorff distance learning, and visual-textual joint relevance learning, etc. [18–23, 25], which have shown good performance in the fields of data mining and image retrieval, may have great potential when used in spatial interpolation applications, especially in combination with other methods. Thus, future studies should aim to investigate the potential of other combination techniques and test their performances under different field scenarios.

## Conclusions

Large spatial variations in soil potassium content within different geographic regions can make it difficult to assess soil properties accurately when there are few data points available. We proposed the use of an ensemble learning model for soil potassium content interpolation, and the method uses valuable information from secondary environment variables for making predictions. Compared with more traditional UK, OK, IDW and OK-Geo interpolation techniques, the proposed EL-SP method not only reduced prediction errors at the study site, but it also produced spatial interpolation maps of soil potassium content that were more reasonable.

Kriging interpolation models usually perform better than IDW and are excellent at least in theoretical analyses [4]. However, in this study the kriging interpolation accuracy was similar (e.g., OK) or worse (e.g., UK) than that of the the IDW method. Other research has found that IDW can be perform better than kriging models when data are isotropic and there are no correlations between the primary variables and the secondary variables [28]. However, the correlations between the primary variables and secondary variables were strong in this study, which suggests that kriging may not always be the optimal model to use for spatial interpolation under a wider range of conditions than previously suspected.

In this study, the interpolation accuracy of the methods that used secondary environmental variables (e.g., OK-Geo and EL-SP) was better than that of the methods that did not use such ancillary information (e.g., UK, IDW, and OK). Thus, interpolation models when combined with appropriate secondary environment variables can effectively improve the simulation accuracy.

limitation of EL-SP is that it has a smoothing effect and that the surface variation is smaller than the ensemble object values. If the ensemble results for an object are smaller than the measured value, the EL-SP results will be lower than the measured value. The current research method used ‘tandem’ ensemble interpolation models, incorporating a global interpolation model for the entire study area, although a simple global model cannot explain the spatial instability of soil properties. In future, we will use ‘parallel’ ensemble interpolation models, based on the different regional characteristics of the study area and with consideration of the problems of simulation scale, to select the appropriate interpolation model integration.

## Supporting Information

S1 Dataset Soil potassium content samples. (XLS)

S1 Fig Slope. (TIF)

S2 Fig Soil types. (TIF)

S3 Fig Geology types. (TIF)

S4 Fig Land use types. (TIF)
