# Supplementary figures and images for: Ensemble Learning for Spatial Interpolation of Soil Potassium Content Based on Environmental Information
Source: PLoS One. 2015 Apr 30;10(4):e0124383. doi: 10.1371/journal.pone.0124383 (PMC4415809; doi:10.1371/journal.pone.0124383)

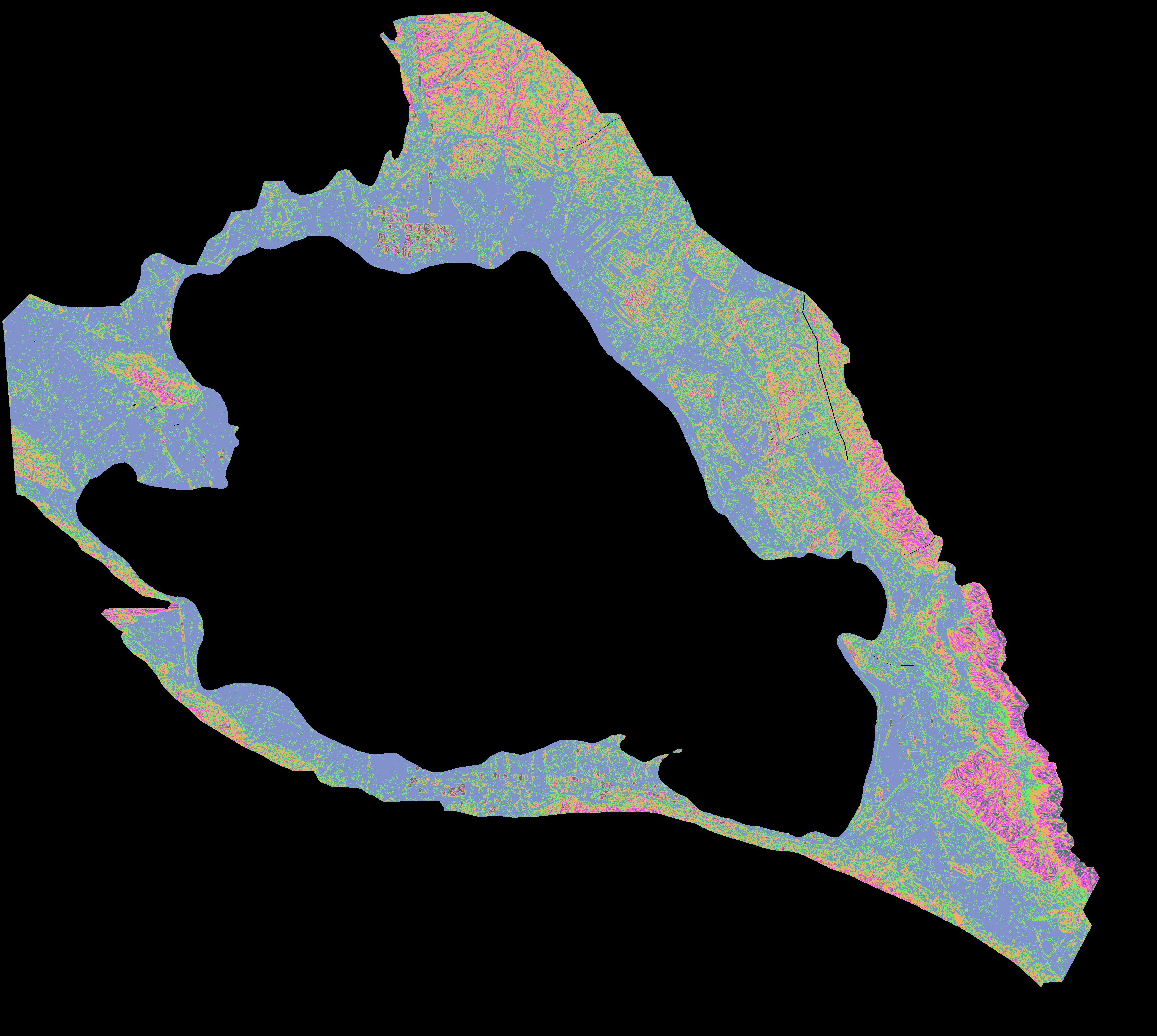

Supplement: S1 Fig — (TIF) [file pone.0124383.s002.tif]

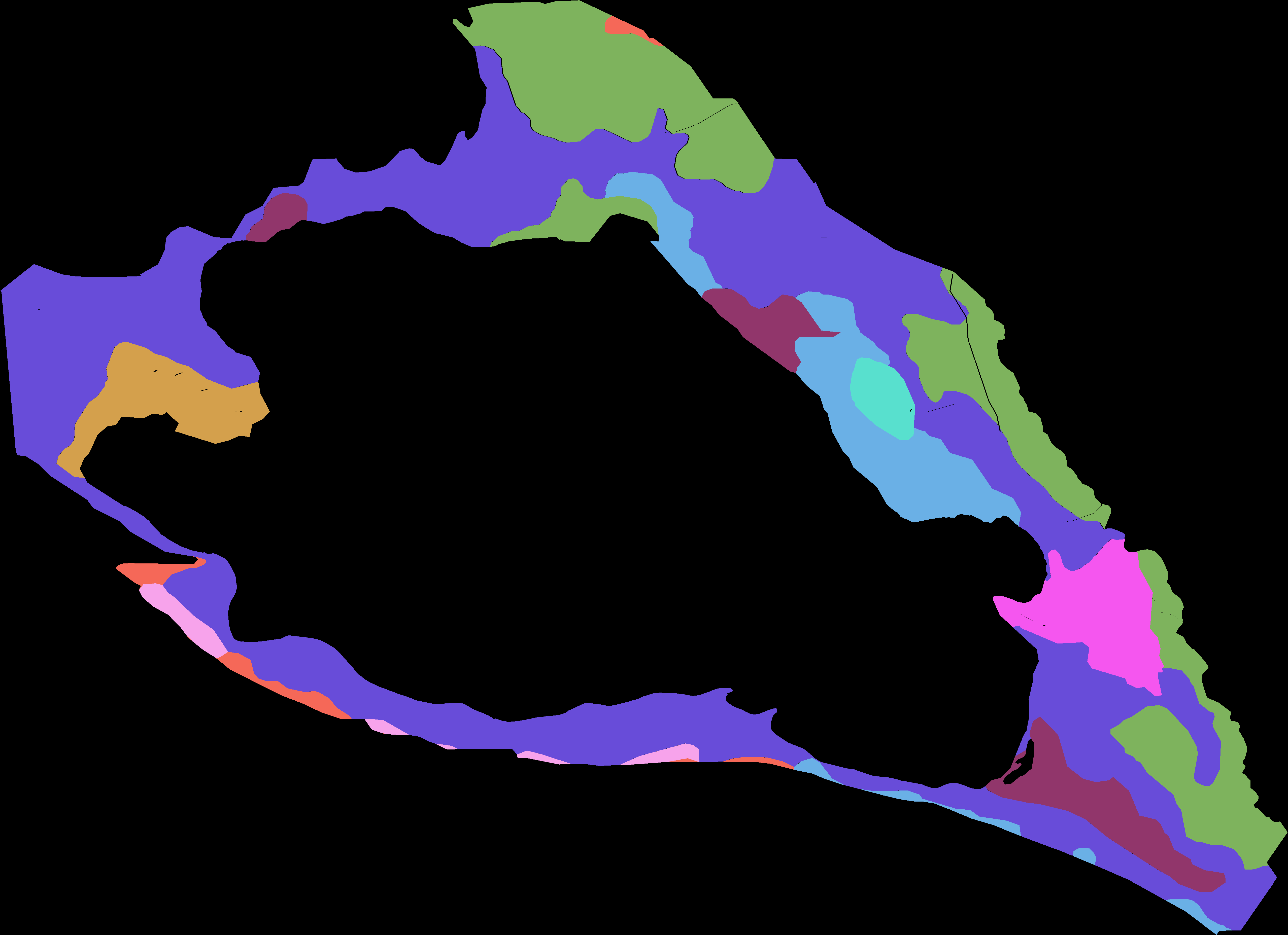

Supplement: S2 Fig — (TIF) [file pone.0124383.s003.tif]

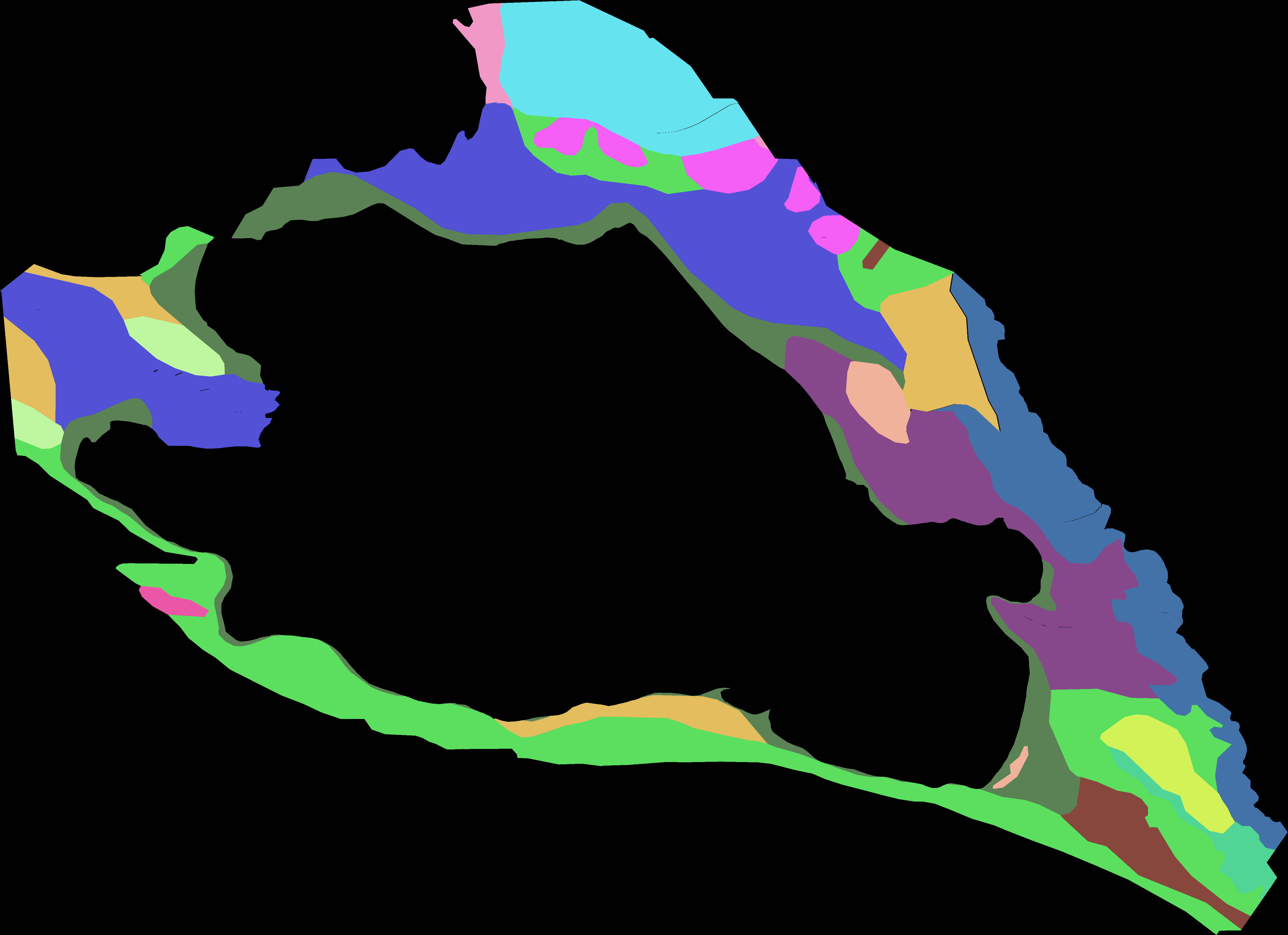

Supplement: S3 Fig — (TIF) [file pone.0124383.s004.tif]

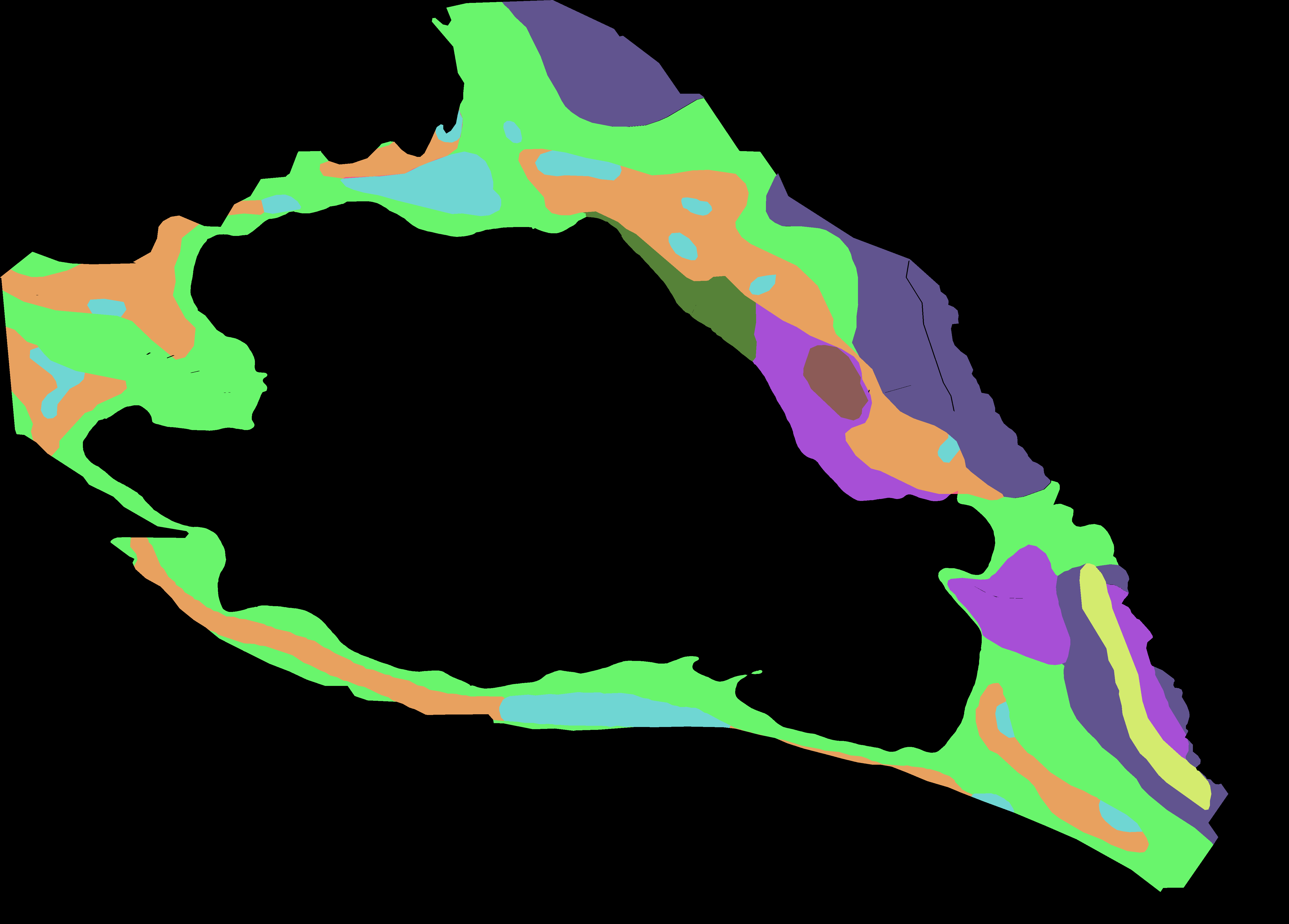

Supplement: S4 Fig — (TIF) [file pone.0124383.s005.tif]
